# Crosstalk between Placental Trophoblast and Decidual Immune Cells in Recurrent Miscarriage

**DOI:** 10.7150/ijms.86533

**Published:** 2023-07-31

**Authors:** Zhenzhen Liu, Yao Tang, Xiaoyue Zhang, Jiangnan Pei, Chengjie Wang, Haiyan Liu, Yi Yu, Shouling Luo, Weirong Gu

**Affiliations:** 1 Department of Obstetrics and Gynecology, Obstetrics and Gynecology Hospital of Fudan University, Shanghai 200011, China.; 2 Shanghai Key Laboratory of Female Reproductive Endocrine Related Diseases, Shanghai 200011, China.

**Keywords:** recurrent miscarriage, maternal-fetal interface, single-cell RNA sequencing, trophoblast cells, immune cells

## Abstract

Recurrent miscarriage (RM) is a pregnancy complication associated with dysregulation of the maternal-fetal interface. We aimed to identify dysfunctional interactions between trophoblast cells and decidual immune cells in RM. We downloaded single-cell RNA sequencing (scRNA-seq) datasets (GSE214607) from the Gene Expression Omnibus (GEO) datasets for further analysis using the R software. The data comprised of paired placental and decidual tissues, including those from patients diagnosed with RM and matched healthy controls. A total of 22976 cells were identified in 11 cell types, including trophoblasts, immune cells, and other cells. We divided trophoblast cells into three types and analyzed their interactions with decidual immune cells. Additionally, we re-clustered NK&T cells and macrophages, identified differentially expressed genes (DEGs), enriched their functions, and compared the cell interactions with trophoblast cells in each cell type. Our single-cell atlas of the maternal-fetal interface revealed alterations in the cellular organization of the decidua and placenta, cell type-specific transcriptome, and cell communication between immune and non-immune cells in RM, which are critical for illuminating the pathophysiology of RM.

## Introduction

Recurrent miscarriage is defined as the consecutive loss of two or more clinical pregnancies and affects approximately 2-5% of couples of reproductive ages 1, 2. Previous studies have revealed that the placenta is central to RM pathophysiology. Abnormal placentation and incomplete formation of the maternal-fetal interface in the first trimester are believed to cause RM 3-6. The placenta is composed of trophoblast cells, including three main epithelial trophoblast types: cytotrophoblast cells (CTBs), syncytiotrophoblast cells (STBs), and extravillous trophoblast cells (EVTs). CTBs are trophoblast stem cells that can fuse and form multinucleated STBs in the floating chorionic villi, which finally establish the intervillous space or labyrinth. Furthermore, CTBs can differentiate into EVTs at the anchoring villi, and EVTs are mainly involved in remodeling maternal spiral arterioles to provide nutrients and oxides to the fetus 7-10. Reduced trophoblast proliferation, excessive trophoblast cell apoptosis, and insufficient trophoblast invasion have been tightly linked to RM development 11. Increasing evidence has revealed that well-orchestrated crosstalk between fetal trophoblast cells and maternal immune cells facilitates the formation of a functional placenta and affects trophoblast cell function during the establishment and maintenance of pregnancy 12. Therefore, it is critical to illustrate the complete atlas of cell communication in the maternal-fetal microenvironment.

Several studies have focused on and illustrated the immune atlas of the human decidua with RM with the development of single-cell sequencing technology 13-16. Notably, Roser et al. reconstructed the crosstalk between trophoblast cells and immune cells at the early maternal-fetal interface in normal pregnancies 17. However, the dysregulation of cell communication in the early maternal-fetal microenvironment in the pathophysiology of recurrent miscarriage remains unknown. In the current study, we used single-cell sequencing data integrating placental and decidual tissues to identify the cell atlas of the maternal-fetal interface and to reveal the crosstalk between trophoblast cells and decidual immune cells in patients with recurrent miscarriage.

## Methods and Materials

### Expression Profile Datasets Selection

The datasets were obtained from the GEO DataSets portal, publicly available at the National Center for Biotechnology Information (NCBI) 18. To identify the cellular and molecular signatures of RM at the maternal-fetal interface, single-cell RNA sequencing data were obtained from the GSE214607 dataset. The data included 16 human first-trimester decidual and villous samples from five normal control (NC) samples and three RM samples, and each decidual and villous sample was collected from the same patients. Four samples were selected for further analysis: GSM6613031 (decidua 6, normal, replicate 1; scRNA-seq), GSM6613036 (villi 6, normal, replicate 1; scRNA-seq), GSM6613041 (decidua 6, RM, replicate 1; scRNA-seq), and GSM6613044 (villi 6, RM, replicate 1; scRNA-seq).

### ScRNA-seq Data Processing and Analysis

#### scRNA-seq Data Processing

Raw sequencing data were processed using Cell Ranger (version2.0.1) 19. The reference index was built using GRCh38 human reference genome assembly (Ensembl 93). Cells with < 600 detected genes or total mitochondrial gene expression > 5% were removed. The obtained matrix was converted into a Seurat object for downstream analysis. We used the FindIntegrationAnchors function provided by Seurat to integrate decidual and villous samples in normal and recurrent miscarriage women, respectively. Downstream analyses, including normalization, shared nearest-neighbor graph-based clustering, differential expression analysis, and visualization, were performed using the standard workflow provided by Seurat (version3.0.3).

#### Cell communication analysis

A systematic analysis of cell communication was based on network analysis and pattern recognition approaches provided by the Cell Chat (version0.01) R package 20. We used a standard workflow to predict the major signaling inputs and outputs of cells and how these cells and signals coordinate functions. Subsequently, we classified the signaling pathways and depicted the conserved and context-specific pathways between RM and NC pregnancies.

#### Cell cycle analysis

To calculate the cell cycle scores, involving G1, S and G2M, in T&NK cells, Cyclone tool of R package scran (version1.18.1) was applied. The results were visualized into T-SNE plot to show the proportion of cell cycle in NC and RM groups.

#### DEGs Analysis and Enrichment Analysis

DEGs were identified using the FindMarkers function 21 (test.use = MAST) in Seurat. P-value < 0.05 and |log2 (fold change) | > 0.58 were set as the threshold for significantly differential expression. The ClusterProfiler R package 22 was used to perform biological functional enrichment analysis of differentially expressed genes using Gene Ontology (GO) and Kyoto Encyclopedia of Genes and Genomes (KEGG). FDR was used to perform multiple test corrections, with the threshold set at p-value < 0.05.

### Statistical analysis

Statistical analysis of single-cell sequencing data was performed in R (version3.5.2) using the Seurat software. The Wilcoxon rank-sum test was used for comparisons between the two groups. Statistical significance was set at p-value < 0.05.

## Results

### Cells in the maternal-fetal interface during early pregnancy

**Figure 1:**
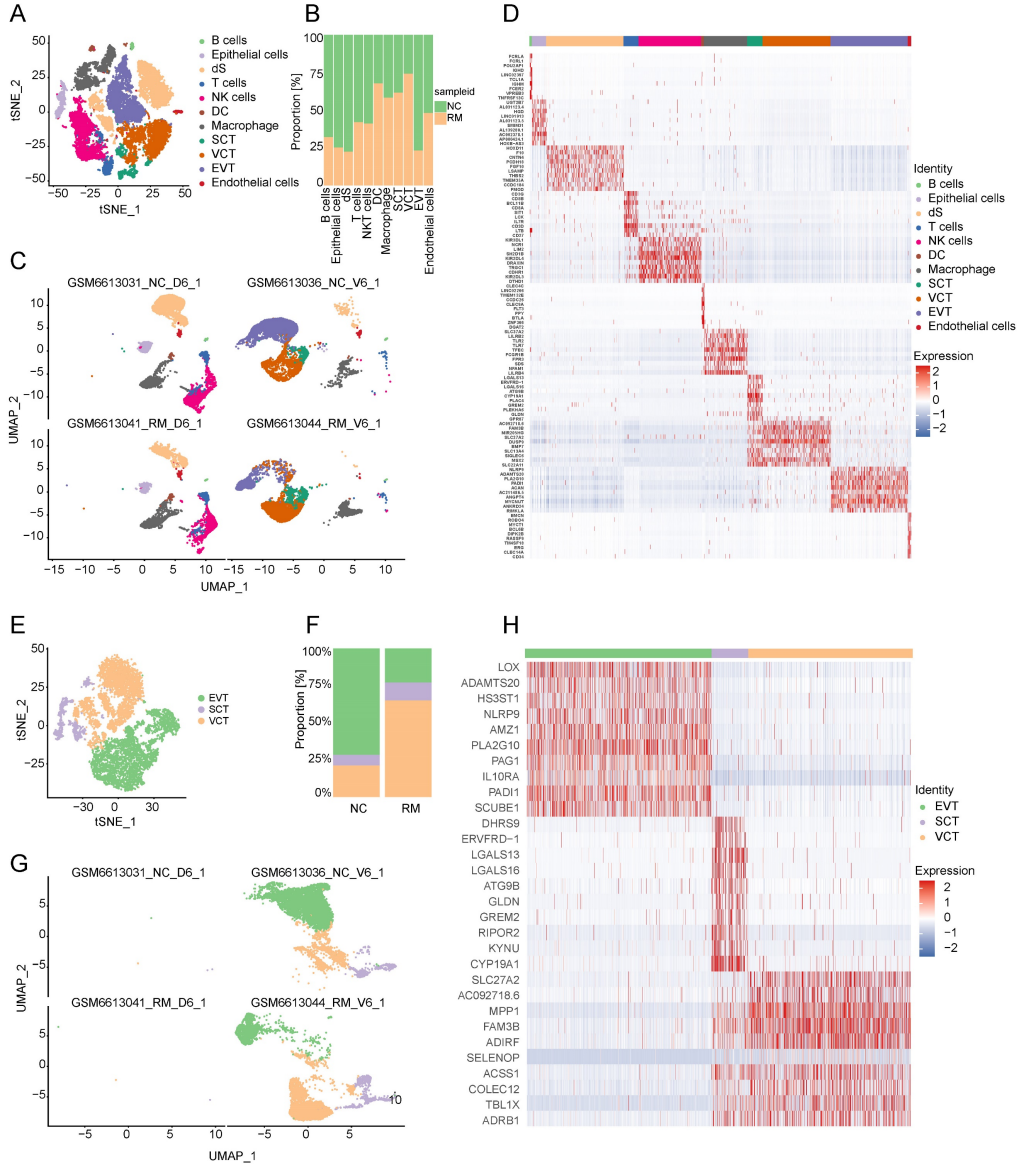
Landscape of all cells from maternal-fetal interface and trophoblast cells during early pregnancy. (A) T-SNE plot showing eleven cell clusters in maternal-fetal interface during early pregnancy. (B) Proportion of eleven cell types in NC and RM groups. (C) UMAP plot showing the proportion of eleven cell clusters in GSM6613031_NC_D6_1, GSM6613036_NC_V6_1, GSM6613041_RM_D6_1, and GSM6613044_RM_V6_1. (D) Heatmap of the top 10 marker genes in all the eleven cell types. (E) T-SNE plot of three trophoblast clusters. (F) The proportion of three trophoblast clusters in NC and RM groups. (G) UMAP plot showing the proportion of three trophoblast clusters in GSM6613031_NC_D6_1, GSM6613036_NC_V6_1, GSM6613041_RM_D6_1, and GSM6613044_RM_V6_1. (H) Heatmap of the top ten marker genes in all three cell types.

To explore the cell composition in placental and decidual tissues from RM and NC patients, we identified 22976 cells (These cells consisted of 7864 cells from normal decidua, 4255 cells from RM decidua, 5490 cells from normal villi, and 5367 cells from RM villi). Eighteen cell clusters and 11 cell types (Figure 1A, C) were identified based on the gene expression profiles of previously reported cell type markers 17, 23, including trophoblast subtypes and several immune cells. The top 10 markers of each cell cluster are shown in a heatmap (Figure 1D).

Interestingly, both the NC and RM groups included the above 11 cell types; however, there were differences in the cell proportions between the two groups. The proportions of dendritic cells (DCs), macrophages, SCTs, and VCTs were higher at the maternal-fetal interface in patients with RM than in patients with NC, whereas the proportions of epithelial cells, decidual stromal cells (dS), B cells, and EVTs were lower in patients with RM (Figure 1B).

### Trophoblast cells

#### Clustering of trophoblast cells in NC and RM pregnancies

Trophoblasts are composed of villous syncytiotrophoblast cells (SCTs), villous cytotrophoblast cells (VCTs), and EVTs. Placental trophoblast dysfunction contributes to significant complications including RM. Therefore, we re-clustered 9693 trophoblast cells into three main trophoblast cell types using the T-SNE and UMAP methods with classical marker genes (Figure 1E, G). The top 10 markers with differential expression among the three trophoblast subtypes are shown in a heatmap (Figure 1H). Additionally, we found that all three trophoblast cell types were present in both the RM and NC groups. The proportion of VCTs was higher in the RM group, the proportion of EVTs was lower in the RM group (Figure 1F).

#### The cell communications between trophoblast cells and other cells

**Figure 2:**
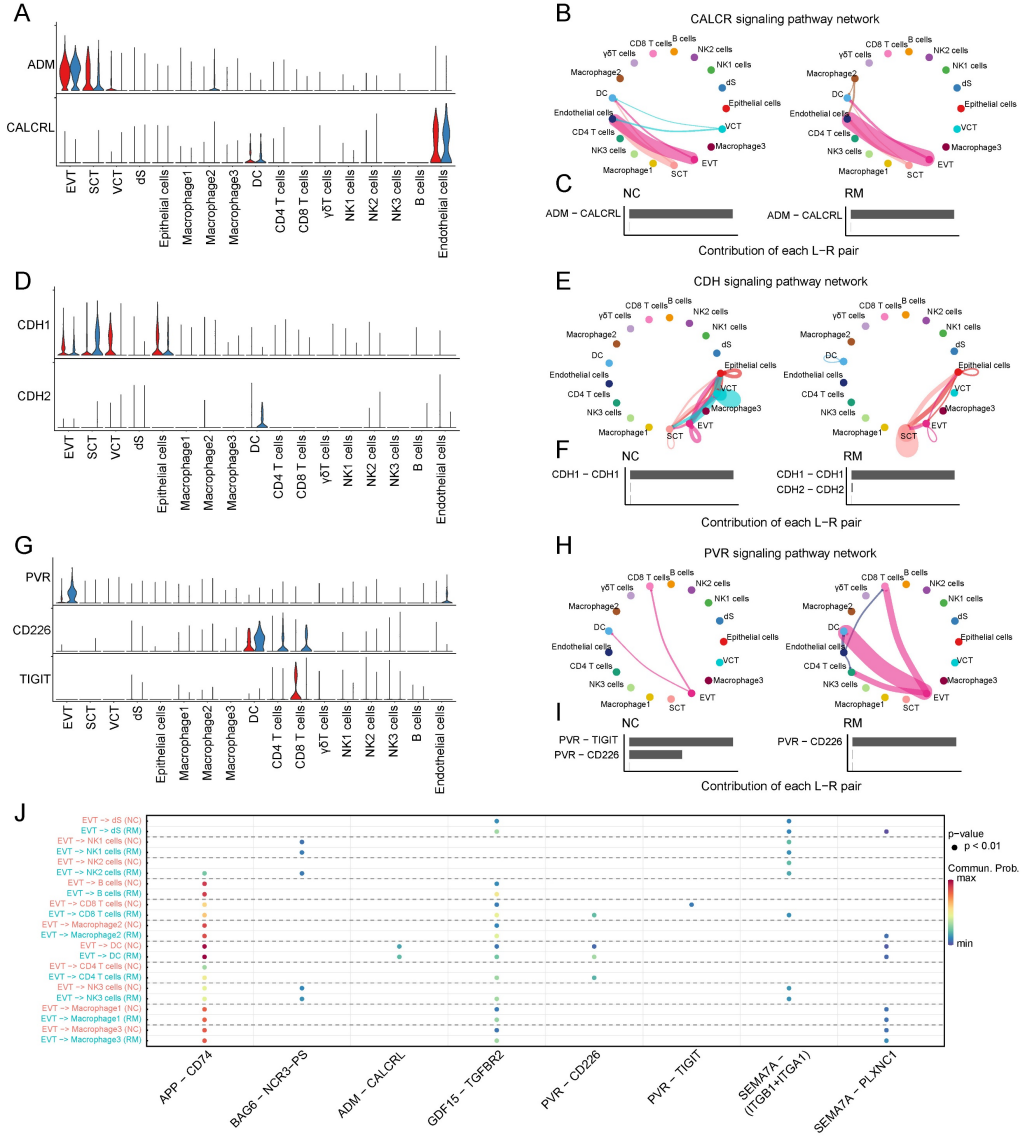
Interaction between trophoblast cells and immune cells. (A) Violin plot showing the expression patterns of signaling genes involved in the CALCR signaling network. Normalized expression levels are shown in a violin plot. Red represents the NC group and blue represents the RM group. (B) Circle plot showing the CALCR signaling pathway network in cells from maternal-fetal interface in NC and RM groups. The thicker the line, the stronger the interaction weights/strength between the two cell types. (C) Comparison of the contributions of the ligand-receptor pairs in the CALCR signaling pathway network between NC and RM groups. (D) Violin plot showing the expression patterns of the signaling genes involved in the CDH signaling network. Normalized expression levels are shown in the violin plot. Red represents the NC group and blue represents the RM group. (E) Circle plot showing the CDH signaling pathway network in cells from maternal-fetal interface in NC and RM groups. The thicker the line represented, the stronger the interaction weights/strength between the two cell types. (F) Comparison of the contributions of ligand-receptor pairs in the CDH signaling pathway network between NC and RM groups. (G) Violin plot showing the expression patterns of signaling genes involved in the PVR signaling network. Normalized expression levels are shown in the violin plot. Red represents the NC group and blue represents the RM group. (H) Circle plot showing the PVR signaling pathway network in cells from maternal-fetal interface in NC and RM groups. The thicker the line represented, the stronger the interaction weights/strength between the two cell types. (I) Comparison of the contributions of ligand-receptor pairs in the PVR signaling pathway network between NC and RM groups. (J) Dot plot exhibited incoming communication patterns of placental trophoblast cells and decidual immune cells.

To identify the interaction between trophoblast cells and immune cells, we performed Cell Chat analysis (Figure 2A-J). ADM, adrenomedullin, which is involved in the CALCR signaling pathway, is mainly expressed by trophoblast cells, particularly EVTs, and its receptor CALCRL is mainly expressed in the membranes of endothelial cells and DCs (Figure 2A-C). Moreover, CDH1 (cadherin 1), associated with cell-cell adhesion, is mainly secreted by trophoblast cells, and the receptor CDH2 (cadherin 2) is expressed in DCs (Figure 2D-F). It was inhibited by the CDH signaling pathway in RM from VCTs to SCTs, EVTs, and epithelial cells, while it was active in RM from SCTs to epithelial cells (Figure 2E). PVR (PVR cell adhesion molecule) is mainly expressed in EVTs, and CD226 is mainly expressed by DCs and T cells. TIGIT (T cell immunoreceptor with Ig and ITIM domains) is mainly expressed by CD8+T cells, which might mediate T cell adhesion and trigger T cell effector functions (Figure 2G-I). Furthermore, it was significantly active in the PVR signaling pathway from EVTs to CD4+T cells, CD8+T cells, and DCs (Figure 2H).

In addition, the amyloid beta precursor protein (APP)-CD74 pair in EVTs and CD4+T cells was active in the RM group, and the GDF15 (growth differentiation factor 15)-TGFBR2 (transforming growth factor beta receptor 2) pair in EVTs and several immune cells including macrophages, NK cells, T cells, and B cells were all active in the RM group (Figure 2J).

### Macrophages

#### Clustering of macrophages in NC and RM pregnancies

**Figure 3:**
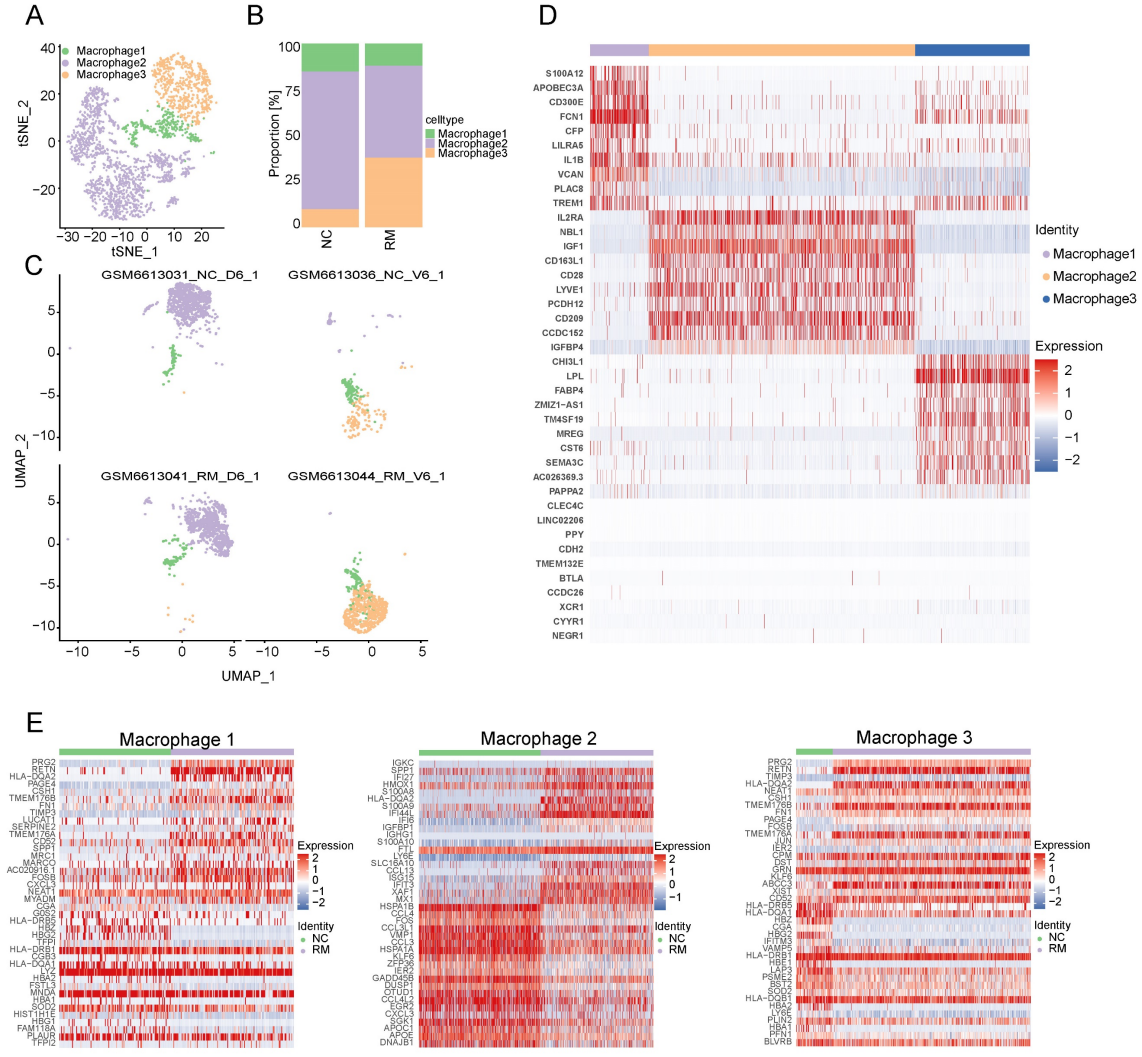
Landscape and functions of macrophages. (A) T-SNE plot showing the three sub-clusters of macrophages. (B) The proportion of three macrophage clusters in NC and RM groups. (C) UMAP plot showing the proportion of three macrophage types in GSM6613031_NC_D6_1, GSM6613036_NC_V6_1, GSM6613041_RM_D6_1, and GSM6613044_RM_V6_1. (D) Heatmap of the top ten marker genes in all four cell types. (E) Heatmap of the top 40 differentially expressed genes (DEGs) in DC, macrophage1, macrophage2, and macrophage3 cells from NC and RM groups.

To explore the function of macrophages in RM, we re-clustered 2777 macrophages into three cell types using T-SNE and UMAP (Figure 3A, C). The top ten marker genes with differential expression between the three macrophage subtypes are shown in a heatmap (Figure 3D). Additionally, we found that all macrophages were present in both the RM and NC groups. The proportion of macrophage 3 was higher in the RM group than in the NC group, and macrophage 3 was mainly located in the villous tissues, whereas macrophage 2 was lower in the RM group (Figure 3B-C). These results were consistent with previous report, which defined macrophage 1 and macrophage 2 as decidual macrophages, while defined macrophage 3 as maternal macrophages 17.

To identify the differences between the three macrophages and their functions in RM, we identified the DEGs in the three macrophage types in the NC and RM groups with a p-value < 0.05, and |log2 (fold change) | > 1.5. Macrophage 1 had 77 DEGs (46 upregulated and 31 downregulated), macrophage 2 had 167 DEGs (83 upregulated and 84 downregulated), and macrophage 3 had 75 DEGs (49 upregulated and 26 downregulated). The top 40 DEGs in macrophages 1, 2, and 3 are shown in Figure 3E. The DEGs of each macrophage type were subjected to GO BP and KEGG analyses (Figure S1A-B). The top 10 GO BP terms and top 10 KEGG pathways are shown in Figure S1A and B, respectively. The results showed that macrophages 1 and 2, as decidual macrophages, are mainly associated with the immune response and inflammation response. Macrophages 3, as maternal macrophage are mainly involving in functions of protecting fetus, such as response to drug, response to mechanical stimulus.

#### The cell communications between macrophages and trophoblast cells

**Figure 4:**
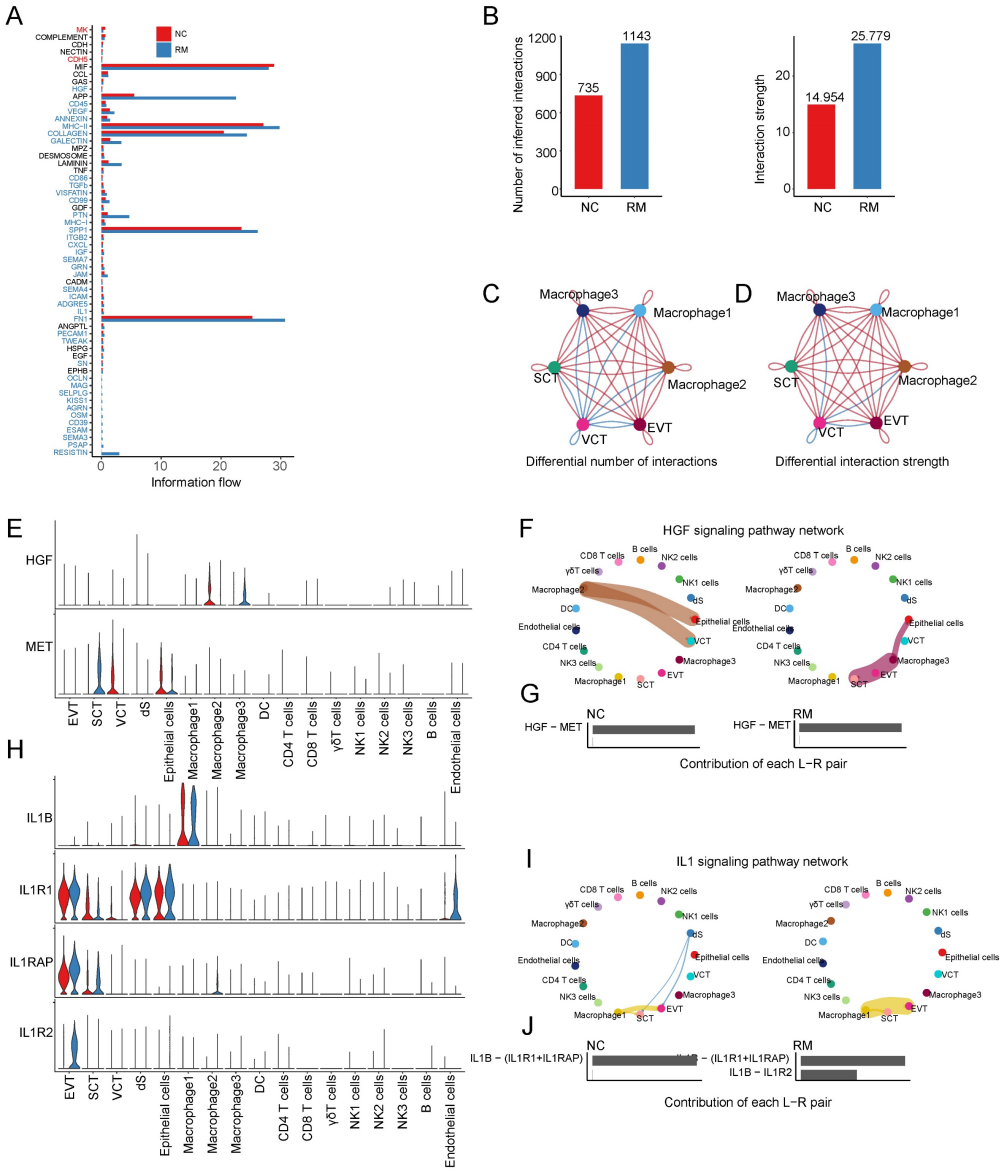
Interaction of macrophages with trophoblast cells. (A) The overall information flow of significant signaling pathways in macrophages in NC and RM groups. The signaling pathways colored red are enriched in NC group, and those colored blue are enriched in RM group. (B) Statistical plot showing the number and strength of significant signaling pathways in macrophages in NC and RM groups. (C) Circle plot showing the differential number of interactions in macrophages between NC and RM groups. The thicker the line represented, the more the number of interactions between the two cell types. (D) Circle plot showing the differential interaction strength in macrophages between NC and RM groups. The thicker the line represented, the stronger the interaction weights/strength between the two cell types. (E) Violin plot showing the expression patterns of signaling genes involved in the HGF signaling pathway network. Normalized expression levels are shown in the violin plot. Red represents the NC group and blue represents the RM group. (F) Circle plot showing the HGF signaling pathway network in cells from maternal-fetal interface in NC and RM groups. The thicker the line represented, the stronger the interaction weights/strength between the two cell types. (G) Comparison of the contributions of ligand-receptor pairs in the HGF signaling pathway network between NC and RM groups. (H) Violin plot showing the expression patterns of signaling genes involved in the IL1 signaling pathway network. Normalized expression levels are shown in the violin plot. Red represents the NC group and blue represents the RM group. (I) Circle plot showing the IL1 signaling pathway network in cells from maternal-fetal interface in NC and RM groups. The thicker the line represented, the stronger the interaction weights/strength between the two cell types. (J) Comparison of the contributions of ligand-receptor pairs in the IL1 signaling pathway network between NC and RM groups.

To identify the cell communication between macrophages and other cells, we used Cell Chat tools for analysis. First, we analyzed the information flow of macrophages (Figure 4A), which showed significant differences between the NC and RM groups. The number of inferred interactions was higher in the RM group than in the NC group (1143 vs. 735), and the interaction strength was also increased in the RM group (25.779 vs. 14.954) (Figure 4B). In addition, we plotted the chord program to reveal the differences in cell interactions between the NC and RM groups (Figure 4C-D).

To identify the comprehensive interaction between macrophages and trophoblast cells, we performed Cell Chat analysis. We found that hepatocyte growth factor (HGF) expression was significantly increased in macrophages, and its receptor MET (MET proto-oncogene, receptor tyrosine kinase) was mainly expressed in SCTs (Figure 4E). The HGF signaling pathway was significantly inhibited from macrophage 2 to epithelial cells and VCTs in the RM group, whereas it was active from macrophage 3 to epithelial cells and SCTs (Figure 4F-G). IL1B (interleukin 1 beta) were mainly expressed in macrophages 1, and their receptor, IL1R1 (interleukin 1 receptor type 1), IL1RAP (interleukin 1 receptor accessory protein), and IL1R2 (interleukin 1 receptor type 2) were all expressed in EVTs (Figure 4H). The IL1 signaling pathway was significantly activated from macrophage 1 to EVTs in the RM group (Figure 4I). The contribution of the IL1B-IL1R2 pair was significantly increased in the RM group (Figure 4J).

### T and NK cells

#### Clustering of T and NK cells in NC and RM pregnancies

**Figure 5:**
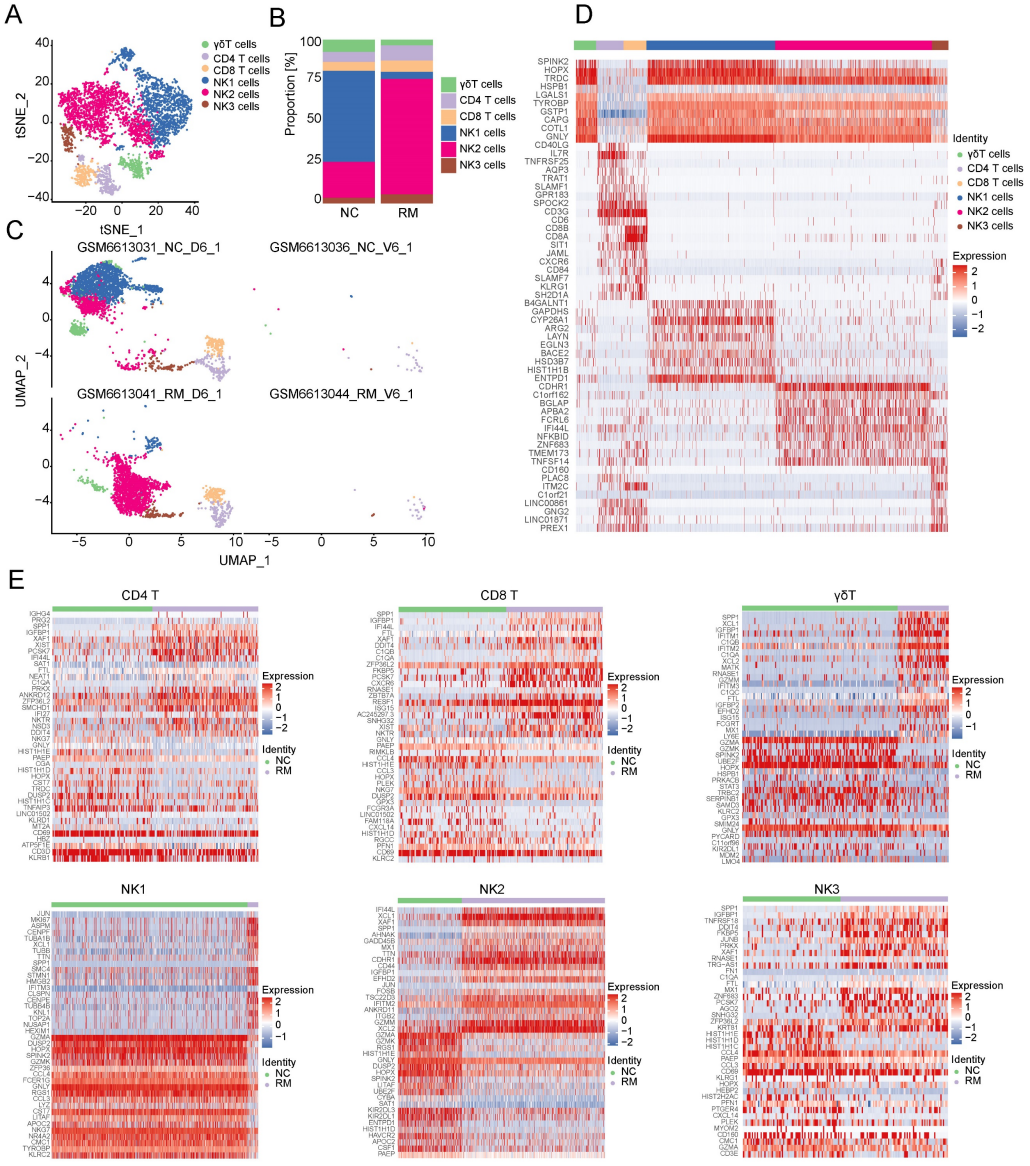
Landscape and functions of T and NK cells. (A) T-SNE plot showing the six sub-clusters of T and NK cells. (B) Proportion of six clusters of T and NK cells in NC and RM groups. (C) UMAP plot showing the proportion of the six types of T and NK cells in GSM6613031_NC_D6_1, GSM6613036_NC_V6_1, GSM6613041_RM_D6_1, and GSM6613044_RM_V6_1. (D) Heatmap of the top ten marker genes for all the six cell clusters. (E) Heatmap of the top 40 DEGs in CD4+T cells, CD8+T cells, γδT cells, NK1 cells, NK2 cells, and NK3 cells between NC and RM groups.

To explore the functions of T and NK cells in RM pregnancies, we re-clustered 896 T cells and 3752 NK cells into 12 clusters using T-SNE and UMAP, respectively (Figure 5A, C). The top 10 marker genes with differential expression between the six T and NK cells and 12 clusters are shown in a heatmap (Figure 5D). Preliminary analysis showed that the upper part was similar to NK cells, whereas the lower part was similar to T cells. Additionally, all six types of T and NK cells were present in both the NC and RM groups. NK2 cells were mainly found in patients with RM, whereas NK1 cells were mainly found in NC patients (Figure 5B).

Owing to the critical role of NK cells in maintaining immune tolerance during pregnancy, subsequent transcriptome analyses were conducted on T and NK cells. To identify the differences between T and NK cells and their functions in RM pregnancies, we identified the DEGs in each cell type in the NC and RM groups with a p-value < 0.05, and |log2 (fold change) | > 1.5. CD4+T cells had 250 DEGs (177 upregulated and 73 downregulated), CD8+T cells had 123 DEGs (86 upregulated and 37 downregulated), and γδT cells had 218 DEGs (138 upregulated and 80 downregulated). NK1 cells had 475 DEGs (381 upregulated and 94 downregulated), NK2 cells had 201 DEGs (132 upregulated and 69 downregulated), and NK3 cells had 117 DEGs (85 upregulated and 32 downregulated). The top 40 DEGs in each cell type are shown in Figure 5E. We obtained the top 10 GO BP terms (Figure S2A) and top 10 KEGG pathways (Figure S2B) using GO and KEGG analyses. These results demonstrate the different biological functions of different T and NK cells.

#### The cell communications between T and NK cells and trophoblast cells

**Figure 6:**
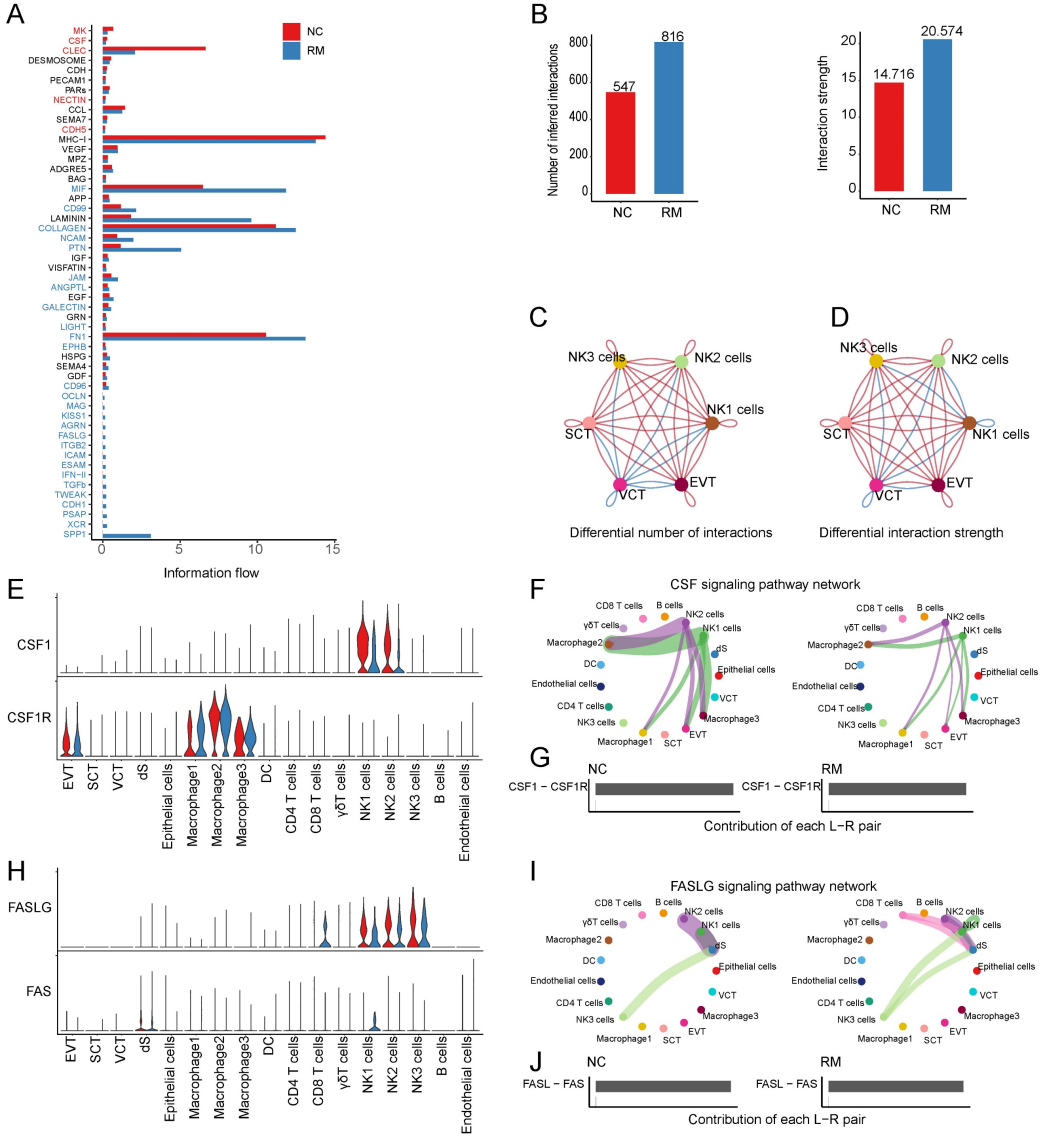
Interaction between NK and T cells and trophoblast cells. (A) The overall information flow of significant signaling pathways in NK and T cells in NC and RM groups. The signaling pathways colored red are enriched in NC group, and those colored blue are enriched in RM group. (B) Statistical plot showing the number and strength of significant signaling pathways in NK and T cells in NC and RM groups. (C) Circle plot showing the differential number of interactions in NK and T cells between NC and RM groups. The thicker the line represented, the more the number of interactions between the two cell types. (D) Circle plot showing the differential interaction strength in NK and T cells between NC and RM groups. The thicker the line represented, the stronger the interaction weights/strength between the two cell types. (E) Violin plot showing the expression patterns of signaling genes involved in the CSF signaling network. Normalized expression levels are shown in the violin plot. Red represents the NC group and blue represents the RM group. (F) Circle plot showing the CSF signaling pathway network in cells from maternal-fetal interface in NC and RM groups. The thicker the line represented, the stronger the interaction weights/strength between the two cell types. (G) Comparison of the contributions of ligand-receptor pairs in the CSF signaling pathway network between NC and RM groups. (H) Violin plot showing the expression patterns of signaling genes involved in the FASLG signaling pathway network. Normalized expression levels are shown in the violin plot. Red represents the NC group and blue represents the RM group. (I) Circle plot showing the FASLG signaling pathway network in cells from maternal-fetal interface in NC and RM groups. The thicker the line represented, the stronger the interaction weights/strength between the two cell types. (J) Comparison of the contributions of ligand-receptor pairs in the FASLG signaling pathway network between NC and RM groups.

To identify cell communication between T and NK cells and trophoblast cells, we used Cell Chat tools for analysis. First, we analyzed the information flow of T and NK cells (Figure 6A), which showed significant differences between the NC and RM groups. The number of inferred interactions was higher in the RM group than in the NC group (816 vs. 547), and the interaction strength increased in the RM group (20.574 vs. 14.716) (Figure 6B). In addition, we plotted the chord program to reveal differences in cell interactions between the NC and RM groups (Figure 6C-D).

**Figure 7:**
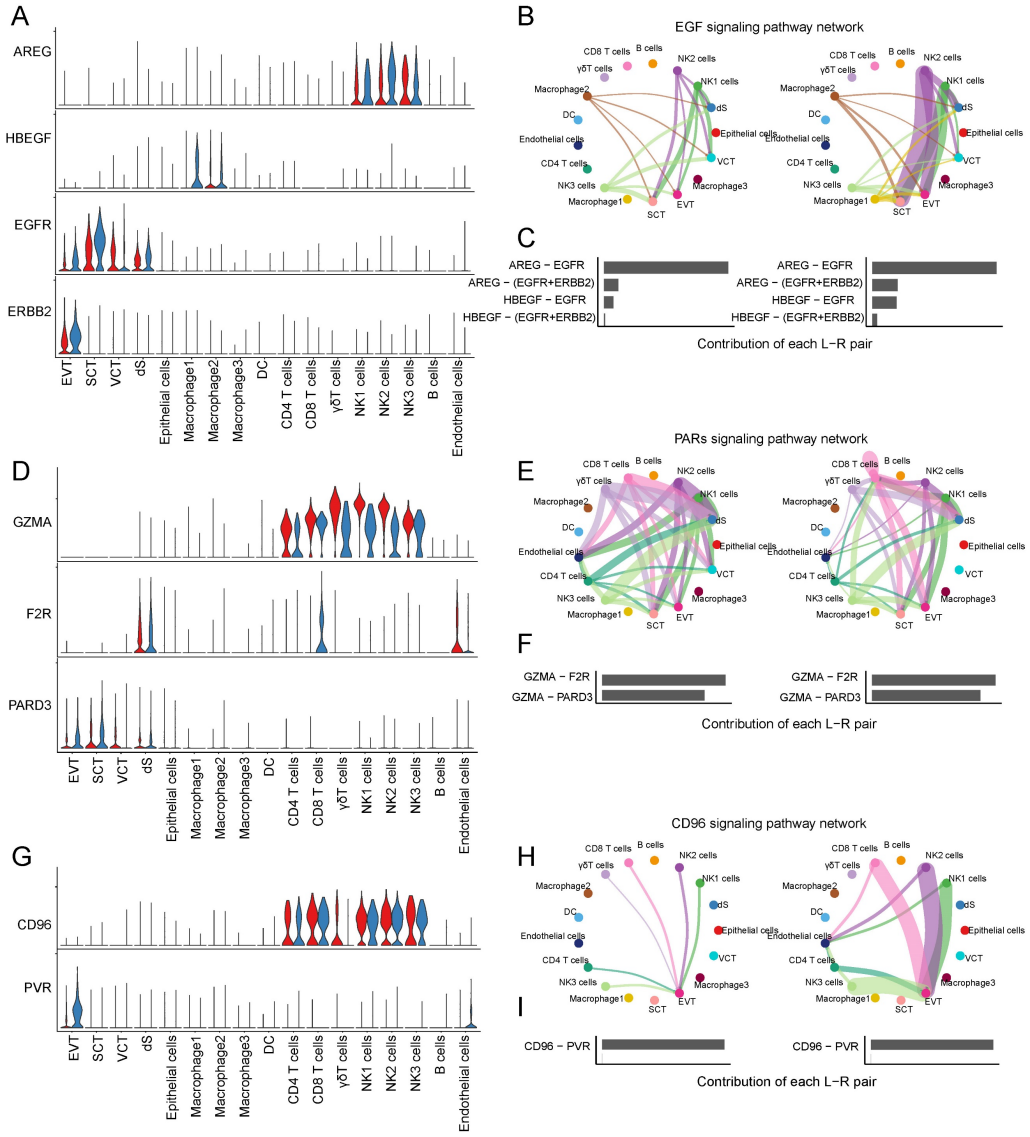
Interaction between trophoblast cells and T and NK cells. (A) Violin plot showing the expression patterns of signaling genes involved in the EGF signaling network. Normalized expression levels are shown in the violin plot. Red represents the NC group and blue represents the RM group. (B) Circle plot showing the EGF signaling pathway network in cells from maternal-fetal interface in NC and RM groups. The thicker the line represented, the stronger the interaction weights/strength between the two cell types. (C) Comparison of the contributions of ligand-receptor pairs in the EGF signaling pathway network between NC and RM groups. (D) Violin plot showing the expression patterns of signaling genes involved in the PARs signaling pathway network. Normalized expression levels are shown in the violin plot. Red represents the NC group and blue represents the RM group. (E) Circle plot showing the PARs signaling pathway network in cells from maternal-fetal interface in NC and RM groups. The thicker the line represented, the stronger the interaction weights/strength between the two cell types. (F) Comparison of the contributions of ligand-receptor pairs in the PARs signaling pathway network between NC and RM groups. (G) Violin plot showing the expression patterns of the signaling genes involved in the CD96 signaling network. Normalized expression levels are shown in the violin plot. Red represents the NC group and blue represents the RM group. (H) Circle plot showing the CD96 signaling pathway network in cells from maternal-fetal interface in NC and RM groups. The thicker the line represented, the stronger the interaction weights/strength between the two cell types. (I) Comparison of the contributions of ligand-receptor pairs in the CD96 signaling pathway network between NC and RM groups.

CSF1 (colony stimulating factor 1) was mainly expressed by NK1 and NK2 cells, and CSF1R (colony stimulating factor 1 receptor) was mainly expressed by EVTs and macrophages (Figure 6E). In RM, the activity of CSF1-CSF1R was inhibited (Figure 6F-G). FASLG (Fas ligand) was mainly expressed by NK and CD8+T cells and the FAS receptor was mainly expressed by dS (Figure 6H). In RM, the FASLG signaling pathway was inhibited from NK2 to dS, from NK1 to dS, and from CD8+T cells to dS (Figure 6I-J). In addition, AREG (amphiregulin) is mainly expressed by NK cells, its receptor heparin-binding EGF-like growth factor (HBEGF) is mainly expressed by macrophages, receptor EGFR (epidermal growth factor receptor) is mainly expressed by trophoblast cells and dS cells, and the receptor ERBB2 (erb-b2 receptor tyrosine kinase 2) is mainly expressed in EVTs (Figure 7A). The EGF signaling pathway was significantly active from NK2 to SCTs and EVTs in patients with RM (Figure 7B). The AREG-(EGFR+ERBB 2), HBEGF-EGFR, and HBEGF-(EGFR+ERBB 2) pairs contributed more to the RM (Figure 7C). Granzyme A (GZMA) is mainly secreted by T and NK cells, its receptor F2R (coagulation factor II thrombin receptor) is mainly expressed by dS and epithelial cells. The receptor PARD3 (par-3 family cell polarity regulator) is mainly expressed by trophoblast cells and dS cells (Figure 7D). The activity of the PARs signaling pathway was significantly altered in patients with RM (Figure 7E). The number of GZMA-PARD3 pairs increased only in the RM group (Figure 7F). CD96 is mainly expressed by T and NK cells, and its receptor PVR is expressed in EVTs (Figure 7G). The CD96 signaling pathway was significantly activated in the RM group, including CD4+T, CD8+T, NK1, NK2, and NK3 to EVTs (Figure 7H-I).

### Decidual stromal cells

**Figure 8:**
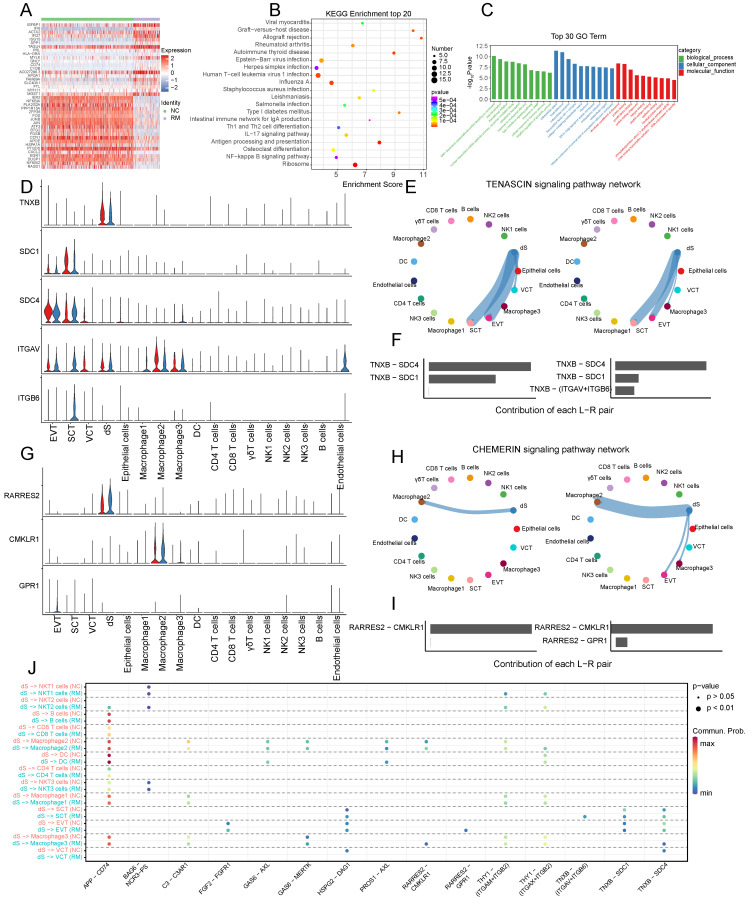
The functions of decidual stromal cells and their interactions with trophoblast cells. (A) Heatmap of the top 40 DEGs in dS cells from NC and RM groups. (B-C) GO and KEGG enrichment for the DEGs in dS cells from NC and RM groups, including the top 20 KEGG pathways (B) and the top 10 GO terms (BP, CC, MF) (C). (D) Violin plot showing the expression patterns of signaling genes involved in the TENASCIN signaling network. Normalized expression levels are shown in the violin plot. Red represents the NC group and blue represents the RM group. (E) Circle plot showing the TENASCIN signaling pathway network in cells from maternal-fetal interface in NC and RM groups. The thicker the line represented, the stronger the interaction weights/strength between the two cell types. (F) Comparison of the contributions of ligand-receptor pairs in the TENASCIN signaling pathway network between NC and RM groups. (G) Violin plot showing the expression patterns of signaling genes involved in the CHEMERI signaling pathway network. Normalized expression levels are shown in the violin plot. Red represents the NC group and blue represents the RM group. (H) Circle plot showing the CHEMERIN signaling pathway network in cells from maternal-fetal interface in NC and RM groups. The thicker the line represented, the stronger the interaction weights/strength between the two cell types. (I) Comparison of the contributions of ligand-receptor pairs in the CHEMERI signaling pathway network between NC and RM groups. (J) Dot plot exhibited incoming communication patterns of dS cells with trophoblast cells.

To further identify the functions of decidual stromal cells, we compared and analyzed the DEGs between the RM and NC groups with a p-value < 0.05, and |log2 (fold change) | > 1.5. A total of 177 DEGs (116 upregulated and 61 downregulated) (Figure 8A) were related to Th1 and Th2 cell differentiation, IL-17 signaling pathway, NF-κB signaling pathway, and ribosome (Figure 8B). The GO terms enriched in these cells were associated with the type I interferon signaling pathway, response to cAMP, cellular response to hormone stimuli, and negative regulation of the apoptotic process (Figure 8C).

To identify cell communication between the dS and other cells, we used Cell Chat tools for analysis (Figure 8D-J). The results revealed that TNXB (tenascin XB) was mainly secreted by dS cells, and its receptors, SDC1 (syndecan 1), SDC4 (syndecan 4), ITGAV (integrin subunit alpha V), and ITGB6 (integrin subunit beta 6), were mainly expressed in trophoblast cells (Figure 8D). The TENASCIN signaling pathway was significantly inhibited from dS to EVTs, SCTs, and VCTs in the RM group (Figure 8E). The contribution of the TNXB-SDC1 pair significantly decreased in the RM group, whereas that of the TNXB-(ITGAV+ITGB6) pair increased in the RM group (Figure 8F). RARRES2 (retinoic acid receptor responder 2) is mainly secreted by dS cells, CMKLR1 (chemerin chemokine-like receptor 1) is mainly expressed in macrophages, and GPR1 (G protein-coupled receptor 1) is mainly expressed in EVTs (Figure 8G). The CHEMRIN signaling pathway was significantly activated from dS to EVTs, macrophage 2, and macrophage 3 in the RM group (Figure 8H). The contribution of the RARRES2-GPR1 pair significantly increased in the RM group (Figure 8I).

## Discussion

In this study, we combined single-cell data from decidual and placental tissues of patients with RM or normal controls to analyze the cell-cell interactions at the maternal-fetal interface. We classified trophoblast cells into three groups, EVTs, SCTs, and VCTs, and analyzed the abnormal interactions between trophoblasts and immune cells in RM using Cell Chat tools. In addition, macrophages and T and NK cells were re-clustered, and their interactions with trophoblast cells, especially the alterations observed in the RM group, were analyzed.

Maternal immune cells, mainly decidual natural killer cells, macrophages, dendritic cells, T cells, B cells, and granulocytes, are derived from the decidual tissues. NK cells represent the main leukocyte population of immune infiltrates in the decidua, accounting for approximately 70% of immune cells at the maternal-fetal interface during early pregnancy 24. Aberrations in the proportion or function of decidual NK cells are closely associated with the occurrence of RM. In our study, NK cells were divided into three main populations, where the proportion of NK1 was lower and the proportion of NK3 was higher in RM group. This finding suggests that NK cells undergo transformation and are involved in the pathological processes of RM.

In addition, crosstalk and interactions exist between NK cells and trophoblasts. Trophoblasts can transmit signals to decidual NK cells through direct contact or by regulating the functions of NK cells by expressing and secreting a variety of cytokines and chemokines 25-27. The abnormal interaction between decidual NK cells and trophoblast cells is associated with pregnancy failure and abortion. Furthermore, previous studies have reported an interaction between NK cells and trophoblast cells. IL-8 secreted by decidual NK cells plays an important role in placental formation and trophoblast invasion 28. Research has shown that inhibition of trophoblast autophagy can significantly inhibit NK cell cytotoxicity and impair trophoblast invasion 29. In the present study, we found numerous abnormal interactions between NK and other cells. For example, CSF1, which is involved in the regulation of cell survival, proliferation, and differentiation, is mainly expressed in NK1 and NK2 and can communicate with SCTs by interacting with CSF1R. In RM, the interaction between the CSF and CSF1R is decreased. This evidence indicates the importance of NK cell interactions with trophoblasts in the pathophysiology of RM.

Decidual macrophages are the second most abundant leukocyte population and comprise 20-25% of all immune cells at the maternal-fetal interface during early pregnancy 30, 31. In the present study, we divided the macrophages into three populations. The proportion of macrophage 3 was significantly higher in RM, and macrophage 3 was mainly present in the placental tissue. The proportion of macrophages 2 was lower in the RM group. Previous studies have shown that macrophages can establish “crosstalk” with trophoblast cells in the maternal-fetal interface microenvironment via a complex cytokine-based connection 32. Additionally, macrophages secrete soluble mediators that regulate the biological behavior of trophoblasts. For example, M2 macrophage-derived G-CSF promotes trophoblast epithelial-mesenchymal transition, invasion, and migration to mediate normal pregnancy 33. In addition, macrophages respond to various factors produced by trophoblast cells to regulate their polarization status, thereby exerting different biological functions, including IL-6 (interleukin 6) 34 and CXCL16 (C-X-C motif chemokine ligand 16) 27. In addition, we found that HGF secreted by macrophages can participate in the pathological mechanisms of RM by binding to MET on the surface of trophoblasts. This evidence suggests that abnormal trophoblast-macrophage communication plays an important role in the pathological process of RM.

The present study also has some drawbacks, we did not validate the differential genes or differential ligand-receptor pairs found in this study. However, for the first time, we have revealed the differences in the interactions between cells at the maternal-fetal interface in RM, which will provide more research directions and a theoretical basis for the study of RM. In addition, decidual and placental tissues were derived from the same patient, which was more convincing.

In conclusion, we elucidated the relationship between trophoblasts and other decidual immune cells using Cell Chat analysis. This study provides a theoretical basis for elucidating the interactions between trophoblasts and immune cells at the maternal-fetal interface in RM, and provides ideas for further studies on RM.

## Supplementary Material

Supplementary figures and tables.

## Abbreviations

RM: recurrent miscarriage
scRNA-seq: single-cell RNA sequencing
GEO: Gene Expression Omnibus
DEGs: differentially expressed genes
CTBs: cytotrophoblast cells
STBs: syncytiotrophoblast cells
EVTs: extravillous trophoblast cells
NCBI: National Center for Biotechnology Information
NC: normal control
GO: Gene Ontology
KEGG: Kyoto Encyclopedia of Genes and Genomes
DCs: dendritic cells
dS: decidual stromal cells
SCTs: syncytiotrophoblast cells
VCTs: villous cytotrophoblast cells
ADM: adrenomedullin
CDH1: cadherin 1
CDH2: cadherin 2
PVR: PVR cell adhesion molecule
TIGIT: T cell immunoreceptor with Ig and ITIM domains
APP: amyloid beta precursor protein
GDF15: growth differentiation factor 15
TGFBR2: transforming growth factor beta receptor 2
HGF: hepatocyte growth factor
MET: MET proto-oncogene, receptor tyrosine kinase
IL1B: interleukin 1 beta
IL1R1: interleukin 1 receptor type 1
IL1RAP: interleukin 1 receptor accessory protein
IL1R2: interleukin 1 receptor type 2
CSF1: colony stimulating factor 1
CSF1R: colony stimulating factor 1 receptor
FASLG: Fas ligand
AREG: amphiregulin
HBEGF: heparin-binding EGF-like growth factor
EGFR: epidermal growth factor receptor
ERBB2: erb-b2 receptor tyrosine kinase 2
GZMA: granzyme A
F2R: coagulation factor II thrombin receptor
PARD3: par-3 family cell polarity regulator
TNXB: tenascin XB
SDC1: syndecan 1
SDC4: syndecan 4
ITGAV: integrin subunit alpha V
ITGB6: integrin subunit beta
RARRES2: retinoic acid receptor responder 2
CMKLR1: chemerin chemokine-like receptor 1
GPR1: G protein-coupled receptor 1
IL-6: interleukin 6
CXCL16: C-X-C motif chemokine ligand 16
