# Supplementary material for: Crosstalk between Placental Trophoblast and Decidual Immune Cells in Recurrent Miscarriage
Source: Int J Med Sci. 2023 Jul 31;20(9):1174–88. doi: 10.7150/ijms.86533 (PMC10416716; doi:10.7150/ijms.86533)
Supplement: Supplementary file 1 — Supplementary figures and tables. [file ijmsv20p1174s1.zip › Supplementary files/Supplemental figure legenes.docx]

**FigureS1.** Functions of macrophages. (A-B) GO and KEGG enrichment for the DEGs in macrophage1, macrophage2, and macrophage3 cells between NC and RM groups, including the top 10 KEGG pathways (A) and the top 10 GO biological process (BP) terms (B).

**FigureS2.** Functions of T and NK cells. (A-B) GO and KEGG enrichment for the DEGs of CD4+T cells, CD8+T cells, γδT cells, NK1 cells, NK2 cells, and NK3 cells in NC and RM groups, including the top 10 KEGG pathways (A) and top 10 GO BP terms (B).

**FigureS3.** (A) Feature plot of TIGIT in NK&T cells. (B) TSNE analysis for cell cycle in NK&T cells split by NC and RM groups. (C) TSNE analysis for cell cycle in NK&T cells. (D) Proportion of three cell cycle phases (G1 phase, G2M phase, S phase) in six NK&T cell subtypes. (E) Proportion of three cell cycle phases in NC and RM groups.
