# Supplementary figures and images for: Crosstalk between Placental Trophoblast and Decidual Immune Cells in Recurrent Miscarriage
Source: Int J Med Sci. 2023 Jul 31;20(9):1174–88. doi: 10.7150/ijms.86533 (PMC10416716; doi:10.7150/ijms.86533)

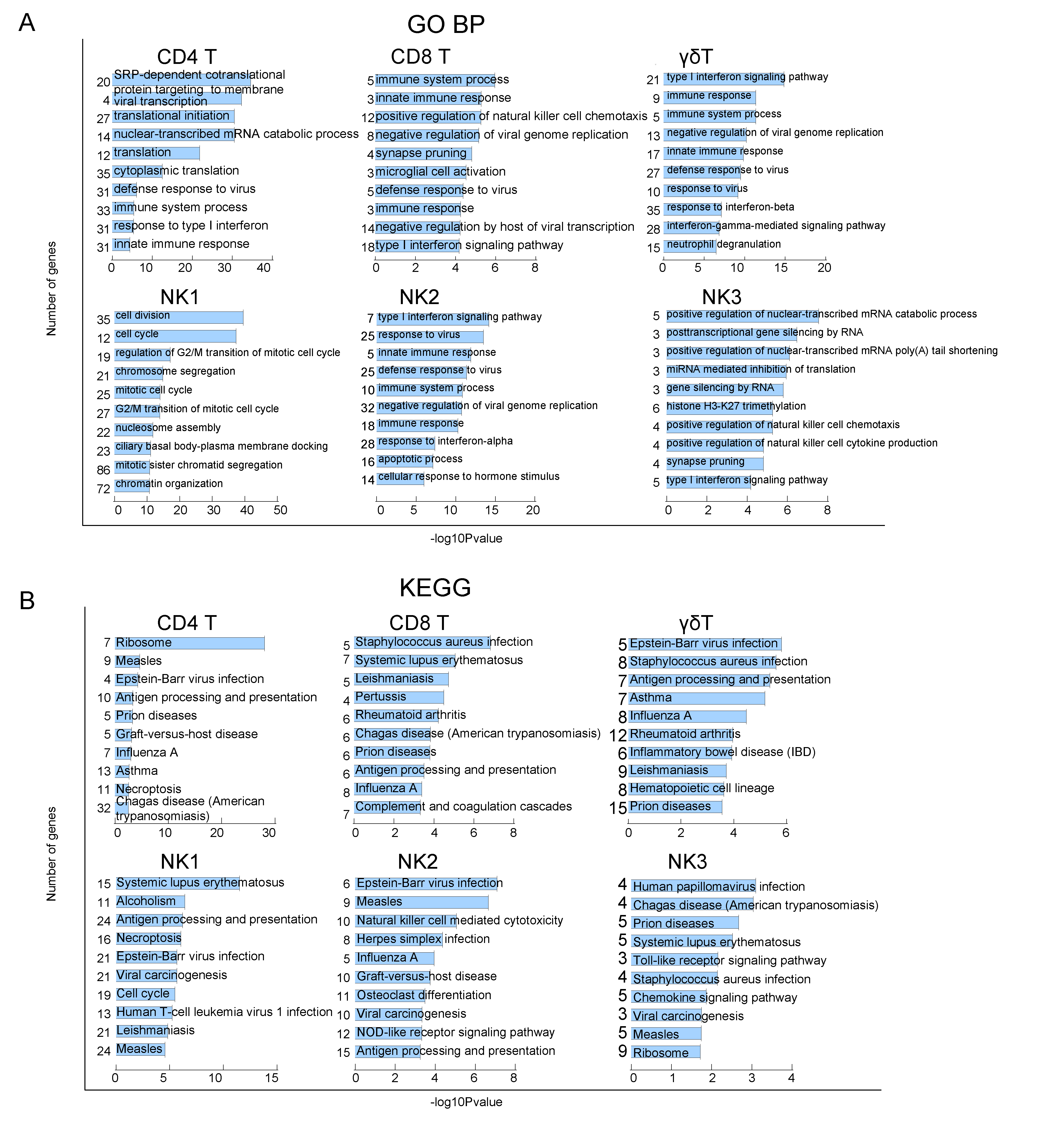

Supplement: Supplementary file 1 — Supplementary figures and tables. [file ijmsv20p1174s1.zip › Supplementary files/FigureS-02.tif]

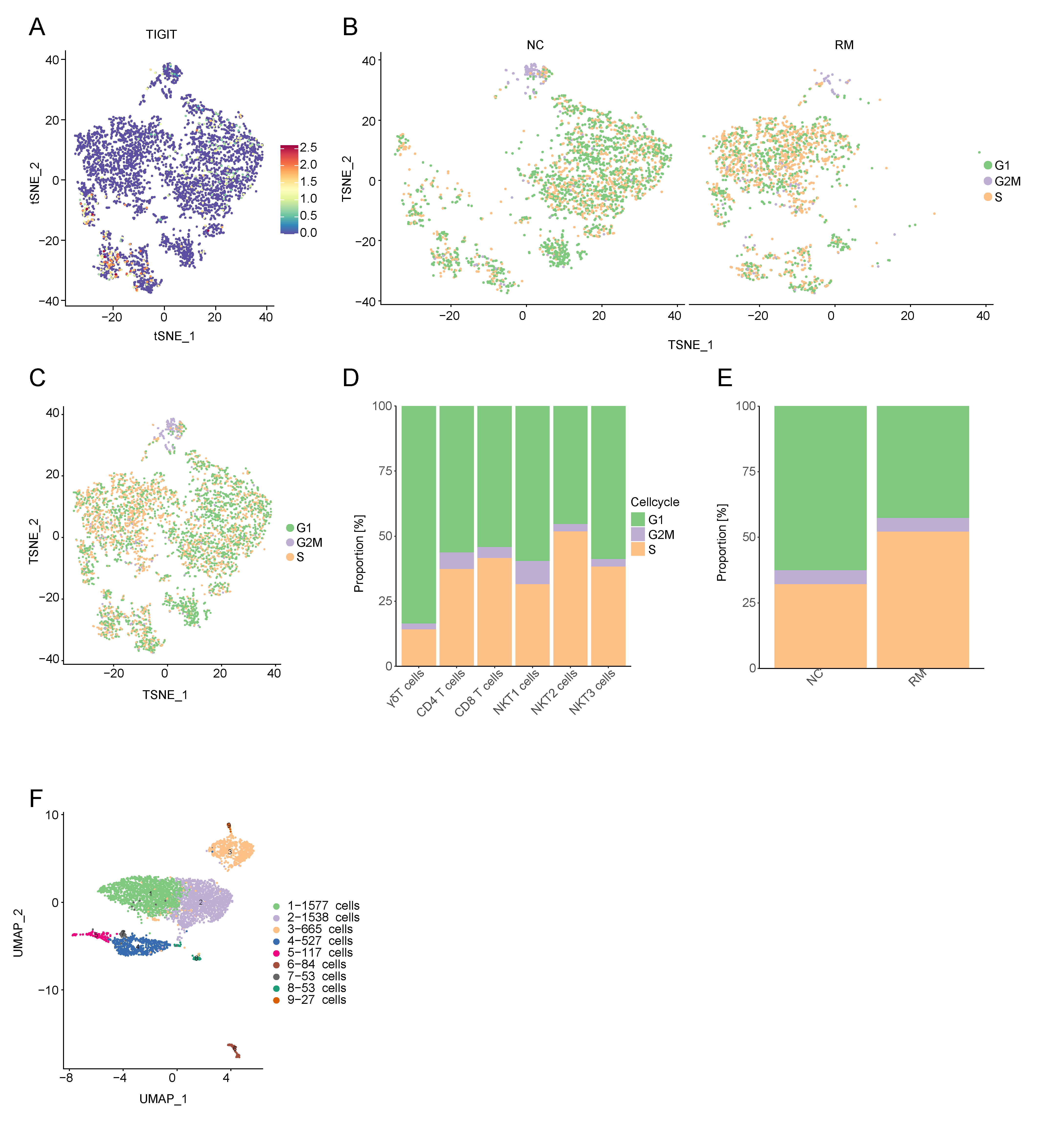

Supplement: Supplementary file 1 — Supplementary figures and tables. [file ijmsv20p1174s1.zip › Supplementary files/FigureS-03.tif]

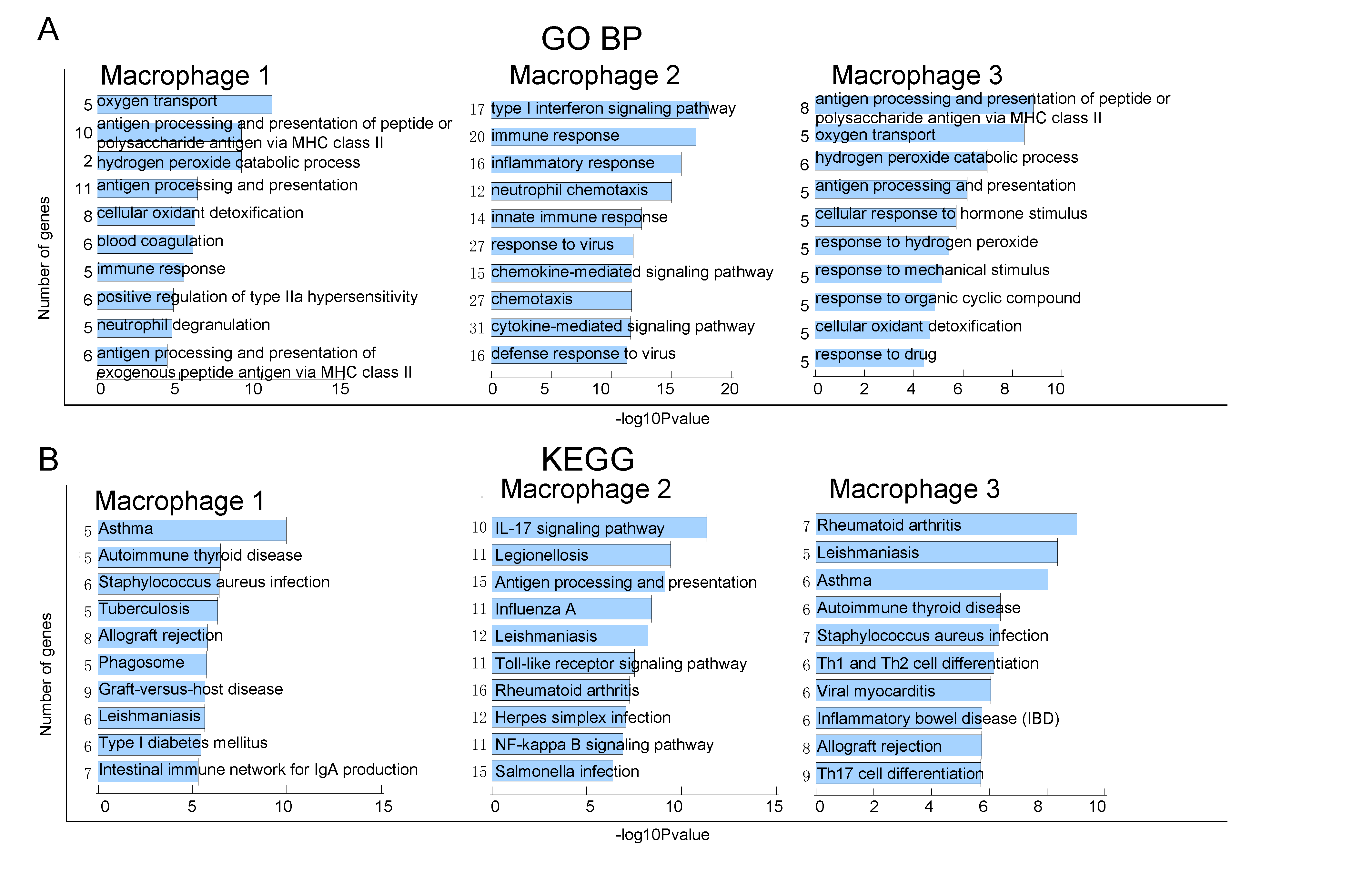

Supplement: Supplementary file 1 — Supplementary figures and tables. [file ijmsv20p1174s1.zip › Supplementary files/FigureS_01.tif]
